# Molecular Threading: Mechanical Extraction, Stretching and Placement of DNA Molecules from a Liquid-Air Interface

**DOI:** 10.1371/journal.pone.0069058

**Published:** 2013-07-31

**Authors:** Andrew C. Payne, Michael Andregg, Kent Kemmish, Mark Hamalainen, Charlotte Bowell, Andrew Bleloch, Nathan Klejwa, Wolfgang Lehrach, Ken Schatz, Heather Stark, Adam Marblestone, George Church, Christopher S. Own, William Andregg

**Affiliations:** 1 Wyss Institute, Harvard University, Boston, Massachusetts, United States of America; 2 Halcyon Molecular, Inc., Redwood City, California, United States of America; 3 Biophysics Program and Wyss Institute, Harvard University, Boston, Massachusetts, United States of America; 4 Department of Genetics, Harvard Medical School, Boston, Massachusetts, United States of America; Université d'Evry val d'Essonne, France

## Abstract

We present “molecular threading”, a surface independent tip-based method for stretching and depositing single and double-stranded DNA molecules. DNA is stretched into air at a liquid-air interface, and can be subsequently deposited onto a dry substrate isolated from solution. The design of an apparatus used for molecular threading is presented, and fluorescence and electron microscopies are used to characterize the angular distribution, straightness, and reproducibility of stretched DNA deposited in arrays onto elastomeric surfaces and thin membranes. Molecular threading demonstrates high straightness and uniformity over length scales from nanometers to micrometers, and represents an alternative to existing DNA deposition and linearization methods. These results point towards scalable and high-throughput precision manipulation of single-molecule polymers.

## Introduction

In solution, a double stranded DNA (dsDNA) double-helix adopts an entropically favourable compact random-coil conformation, and a single stranded DNA (ssDNA) molecule, having a smaller persistence length, is more compact still [1]. By applying tension to the strand, a DNA molecule can be stretched into an entropically unfavorable, elongated state.

The first method developed for mechanical elongation of DNA involved stretching fibres of its precipitated sodium salt [2] in air, and a number of techniques for stretching DNA from or in solution have been developed since. Existing bulk methods for stretching DNA include molecular combing [3], [4], [5], [6], transfer printing [7], and shear-induced stretching in micro- and nano-channels [8]. These techniques have been used to prepare DNA for sequence motif mapping and to characterize chromosomal abnormalities [9], [10]. Molecular combing - where DNA molecules are elongated on compatible surfaces by the action of a receding meniscus - combined with transfer printing and electron microscopy, shows promise as a tool to enable direct reading of genetic and epigenetic information from stretched DNA molecules [11]. Existing single-molecule manipulation techniques, such as optical and magnetic tweezers [12], [13], atomic force microscopy [14], and micro-needle manipulation [15], have had success characterizing the behaviour of DNA and other bio-polymers under tension and torsion, as well as their interactions with other biomolecules [16], [17].

Existing methods for stretching DNA have inherent limitations. Bulk methods such as molecular combing or transfer printing require either liquid (buffer) or solid (stamp) contact with the substrate, both of which are incompatible with sensitive or fragile surfaces. Additionally, stretching in molecular combing is sensitive to both the surface and the buffer [18], [19], [20]. A method to elongate DNA molecules while avoiding bulk contact of the carrier with the substrate is desirable because it circumvents these constraints. While single-molecule manipulation techniques can stretch DNA molecules independent of a surface, existing single-molecule methods do not enable the transfer of stretched molecules to a target substrate and are generally slow.

To overcome these limitations, we have developed an efficient tip-based method to mechanically stretch DNA molecules and deposit them onto a surface [21]. In this novel method, stretching is accomplished by mechanically pulling DNA segments from a droplet using a pulled-glass micro-needle and suspending them in air between a liquid-air interface and the micro-needle tip. The segments can be deposited onto a dry substrate in a separate step, avoiding bulk contact of the carrier medium with the deposition surface. We call this method “molecular threading.”

## Results

### I. Construction and operation of a molecular threading device for DNA

Molecular threading works by extracting one or more DNA molecules from a liquid buffer into air using the tip of a chemically-passivated pulled-glass micro-needle. The passivation layer, created by immersing the needle-tip in a suspension of Poly(methyl methacrylate) (PMMA) in acetone, provides simultaneous DNA affinity and fluid hydrophobicity. Threading proceeds by inserting the passivated needle-tip into a droplet of DNA-containing buffer solution. The needle is then extracted from the droplet, and one or more DNA molecules bound to the needle are pulled through the liquid-air interface of the droplet. Surface tension acting on the DNA at the liquid-air interface provides a restoring force that stretches the DNA segment suspended in air. The stretched DNA can be placed on a substrate positioned underneath it by lowering the needle-tip to the substrate surface, anchoring the molecule by weak forces to the substrate in a stretched configuration.

**Figure 1:**
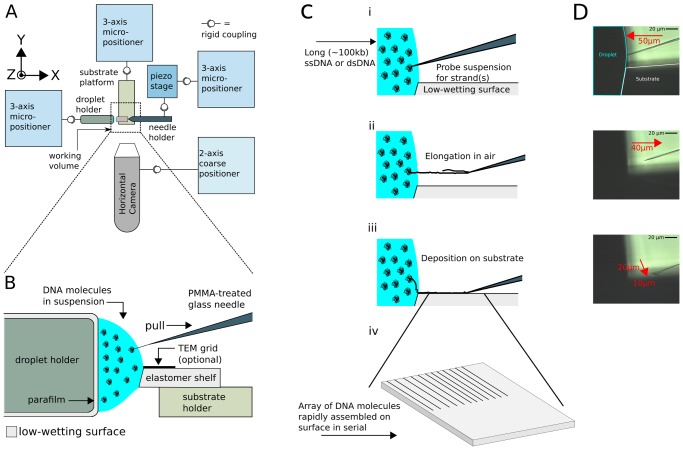
Molecular threading apparatus. (**A**) Device for stretching and positioning DNA molecules extracted at a liquid-air interface. A set of three 3-axis micro-positioners are used for coarse alignment of a DNA-binding tip, a target substrate, and the droplet containing a DNA suspension. Horizontal and vertical cameras are used to monitor the process (vertical camera not shown). (**B**) Illustration of the work area for “spider” threading. The droplet is immobilized between two low-wetting surfaces and a piezo-controlled tip is then moved between the surface of the suspension and the adjacent substrate. (**C**) Molecular threading at a liquid-air interface. (**i**) Needle tip penetrates into DNA suspension, binding to one or more strands (collectively called a “thread”). (**ii**) Needle tip extracts from the suspension parallel to substrate surface. The restoring force of the meniscus places the bound thread in tension. (**iii**) Needle tip is brought into direct contact with the target surface, transferring the strand to the substrate and the needle. (**iv**) A lateral translation of the needle after each extraction permits the creation of an array of linearized DNA. (**D**) Images of the needle, droplet, and substrate, taken under magnification, and corresponding to the illustrations in (C).

We designed an apparatus to independently actuate the three main components in threading (glass micro-needle, DNA suspension droplet, and target substrate) and enable real-time monitoring of the process. A schematic representation is shown in **Figure 1A**, and photographs of the apparatus are shown in **Figures S1** and **S2**. Each component is rigidly attached to an independent long-range three-axis micropositioner for coarse alignment. The motion of the needle must be controlled with higher precision by a secondary computer-controlled piezoelectric nanopositioner mounted on a coarse micropositioner. The components are aligned inside the nanopositioner working volume (120 µm×120 µm×120 µm) using cameras aimed in the -Z (substrate normal) and +Y (perpendicular to needle) axes. Various geometries are possible: the DNA-containing droplet and substrate may be placed in contact with each other or separated by a gap, and the needle may be configured to either contact the substrate during its stroke or avoid contact. In the latter no-contact case, the needle is extended past the edge of the substrate along the needle (X) axis and then brought below the substrate edge in the Z-axis to simultaneously shear the strand while depositing it.

Substrate and droplet separation is advantageous in some applications; however we have typically found straighter deposited strands when the substrate is partially contacted by the droplet. This mode is shown in **Figure 1B**, which depicts the working volume of the instrument in the partially-contacted configuration. The droplet is immobilized between two low-wetting surfaces, and both sides of the liquid-air interface as well as the substrate surface are within the range of fine motion of the micro-needle tip. We call this configuration “spider threading” since the threaded strands extend from a partially combed region like the legs of a spider. Spider threading has certain advantages: (1) reduced local evaporation rate since only part of the droplet is exposed, (2) straighter strands due to more consistent meniscus shape and reduced evaporation rate, and (3) additional deposition of a partially-combed region with high-density fibrous bundles which can serve as fiducials during imaging for identifying the maximum extent of the meniscus. **Figure 1C** illustrates the steps of the optimized “spider” molecular threading process:

- The nanopositioner moves according to a pre-programmed path, causing the tip of the needle to penetrate the droplet meniscus and into the liquid.
- The positioner then reverses direction, extracting the needle from the droplet by a pre-programmed distance (extraction distance). Any DNA molecules attached to the needle, collectively called a “thread”, are mechanically separated from the droplet and its contents, and suspended in tension above the substrate between the withdrawing tip and the liquid-air interface by the restoring force of the meniscus. The number of attached and/or intertwined DNA molecules attached to the tip is not known until subsequent interrogation, e.g., via high-resolution imaging such as TEM or AFM. The thread remains normal to the droplet surface during manipulation: this effect is shown in **Video S1** for a multi-strand thread large enough to be visible via light microscopy. As the thread exceeds its extraction limit and is pulled out of the meniscus, it un-stretches but remains bound to the needle. By reintroducing the needle to the droplet, the meniscus force on the DNA is restored and it can be re-stretched by the needle action. This indicates that if the DNA remains bound to the needle tip the stretching process is reversible.
- When the tip is brought into contact with the substrate, the thread is transferred and immobilized onto the substrate surface. This contact with the surface also cleans the tip, freeing it to repeat the threading process.
- By translating the tip across the substrate transverse to the previously-deposited threads (Y-axis), an array of adjacent, approximately parallel threads can be created with an operator-defined lateral pitch.

The process may be repeated indefinitely over the same substrate area, producing arbitrarily dense arrays of straight parallel threads.

### II. Properties of threaded DNA

**Figure 2:**
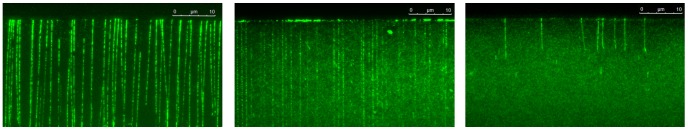
Threading of single and double-stranded DNA. Various DNA types are compatible with molecular threading. (**A**) Concatamers of m13mp18 ssDNA prepared by rolling-circle amplification, extracted to 40 µm. (**B**) Double-stranded genomic T4 GT7 bacteriophage DNA, extracted to 40 µm. (**C**) Double-stranded *λ* bacteriophage DNA, extracted to 10 µm. All samples were threaded onto different elastomer substrates and stained with intercalating dye YOYO-1 prior to deposition.

Molecular threading is a high-throughput technique: the motion of the piezoelectric nanopositioner sets the speed of threading, which is able to exceed 10 Hz. To assess macroscale properties, we prepared arrays (typically 50–100 threads) from single and double stranded DNA by threading on low-wetting elastomer shelves using a nominal thread-to-thread pitch of 1 µm (**Figures 2** **–** **3**). The threaded DNA was stained with YOYO-1 intercalating dye and imaged by fluorescence microscopy. For investigations at higher resolution, we conducted electron microscopy (EM) imaging of threaded modified ssDNA strands stained with a thymidine-selective osmium tetroxide 2-2′ bipyridine (osbipy) contrast-enhancing label (**Figures 4** **–** **5**). The synthesis and labelling kinetics of this agent have been discussed in previous work [22].

**Figure 3:**
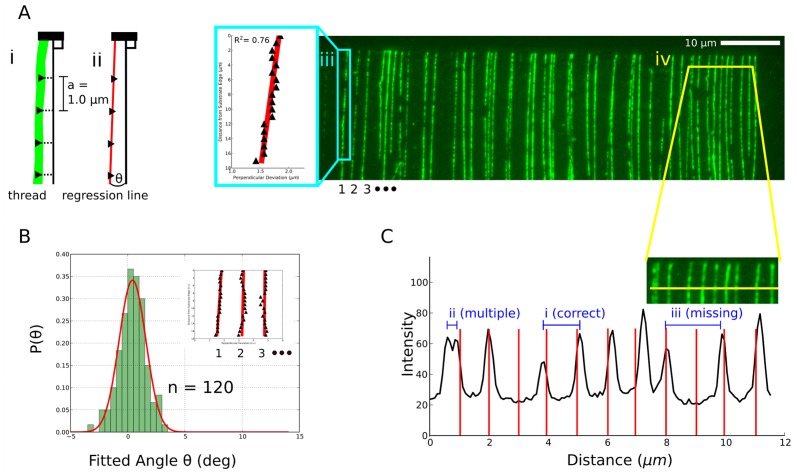
Characterization of threaded DNA arrays by fluorescence microscopy. (**A**) **Left:** Scheme for determining the angle of a thread. (**i**) The horizontal position of a fluorescent trace of a YOYO-1 stained thread is measured at a series of vertical positions in 1.0 µm increments. The vertical axis is defined to be perpendicular to the edge of the target substrate. (**ii**) A regression line is fit to the horizontal position measurements and used to define the angle of the thread with respect to the vertical axis. **Right:** Wide-field fluorescent image of an array of YOYO-1 stained threads of ssDNA molecules generated by rolling circle amplification of m13mp18 ssDNA. (**iii – blue box**) Example of the use of linear regression to measure the angle of the left-most thread. (**iv – yellow cross-section**) Horizontal profile spanning 10 individual threads. (**B**) Histogram of angles determined for 120 threads compiled from two arrays prepared at different locations on an elastomer substrate. **Inset:** regression curves for three individual threads from the image in (A). (**C**) Illustration of successful and failed threading across 11 needle insertions (area sampled from (A)). (**i**) Successful threading: two adjacent threads are present with the desired spacing determined by the programmed lateral translation of the needle after each insertion. (**ii**) Two intertwined threads captured by the needle on a single insertion. (**iii**) No thread is present at the corresponding needle insertion position.

**Figure 4:**
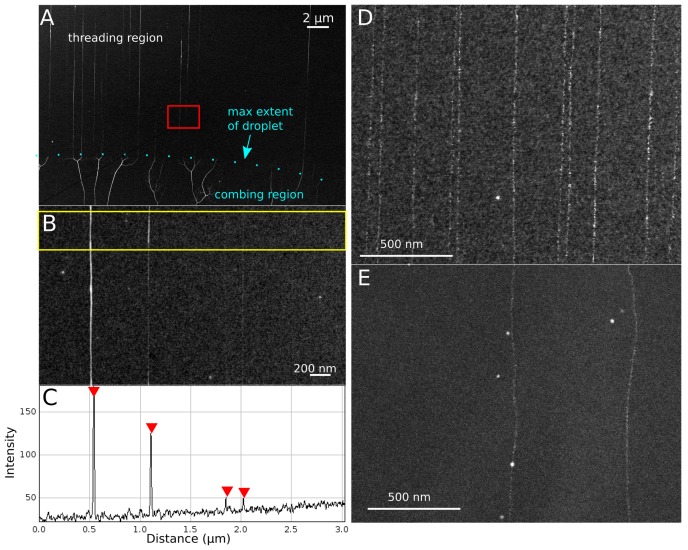
SEM characterization of threaded DNA. All images were taken on a Hitachi S-5500 high-resolution transmission SEM (∼1 nm resolution), operated at 30 kV, with 50 µA maximum post-flash current. (**A**) Synthetic ssDNA modified with EM-dense labels to enhance contrast against 5 nm thick semiconductor support films. The deposition pattern indicates the behavior of DNA under the action of a receding meniscus compared to the molecular threading process. (**B**) High resolution image of a section of four deposited threads ((A) – red box). (**C**) Intensity profile for a cross section of ((B) - yellow box). The non-homogeneous contrast between threads is likely explained by variability in the number of ssDNA molecules making up each thread, while non-homogeneous contrast between different regions of the same thread is likely explained by secondary structure. (**D & E**) Comparison between (D) molecular threading and (E) molecular combing of synthetic ssDNA, modified with a thymidine-specific label osmium 2-2′-bipyridine (osbipy), onto 5 nm thick amorphous Si substrates, with contrast around strand backbone enhanced for publication clarity.

**Figure 5:**
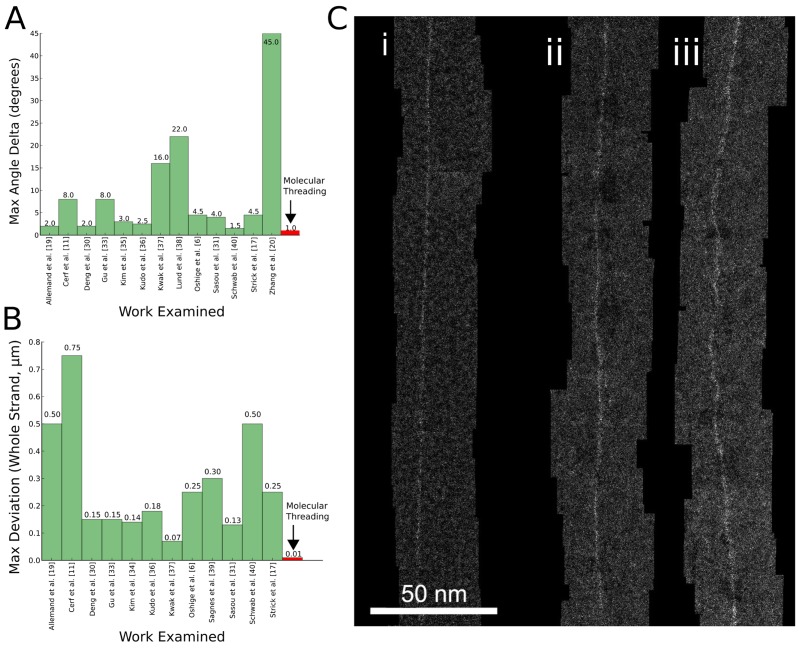
Advantages of molecular threading over combing. (**A**) Comparison of maximum angular deviation between threading and combing examples from the literature. (**B**) Comparison of maximum lateral deviation in µm for similar strand length. (**C**) Comparison between molecular threading (**i**) and combing (**ii–iii**) at high resolution, with synthetic DNA base-specifically labelled with targeted EM-dense contrast agents placed onto 5 nm thick amorphous Si substrates. In general, threaded strands are much straighter than combed strands. Combed strands can be quite straight in sections on the order of 100 nm (**ii**), but over larger distances large lateral deviations can occur in a different region along the same strand (**iii**). Composite images are 230 nm sections of imaged strands several µm long obtained on a Nion UltraSTEM 100 atomic-resolution STEM (∼1 Å resolution) retrofitted to do automatic stage movement. The UltraSTEM is operated at 60 kV, with 100 pA current measured on the Ronchigram.” Contrast around strand backbone is enhanced for publication clarity.

### III. Applicability to multiple DNA and substrate types

Molecular threading reliably produces arrays of parallel threads on PDMS substrates using both single-stranded and double-stranded DNA, and is compatible with a variety of DNA and buffer combinations. After staining the substrate surface with an intercalating dye these arrays can be detected using fluorescence microscopy (**Figure 2**). **Figure 2A** shows long (∼380 kbp) m13mp18 ssDNA concatamers prepared using rolling circle amplification [23] that were threaded with an extraction distance of 40 µm. The 167 kbp double-stranded viral genome of bacteriophage T4 GT7 was also found to have similarly high deposition efficiency at the same extraction distance (**Figure 2B**). However, the shorter, double-stranded viral genome of *λ* bacteriophage (48.5 kbp, approximately 16 µm crystallographic length) did not thread successfully at this extraction distance: only at a shorter extraction distances of less than 20 µm could DNA be detected (**Figure 2C**). This result indicates that the extraction distance for a given DNA length cannot exceed a critical value, and any attempt to extract a thread beyond this distance causes threading yields to drop precipitously. This is expected, since after extraction a sufficient length of excess DNA must remain in the droplet so as to allow controlled stretching and placement of the thread by the micro-needle.

Arrays of parallel threads may also be placed onto many types of unsupported thin films, which remain intact even after repeated contact with the tip of the needle. Commercially-available 5 nm thick Si and SiN TEM grids from TEMWindows and Dune Sciences are compatible with threading, and floated carbon supports as thin as 2.5 nm can be threaded on if the droplet and needle do not contact the film. For tougher semiconductor film types, the DNA-containing droplet can be placed directly in contact with the bulk support adjacent to the film window that will be used to support threaded DNA (**Figure S3**). Strands are deposited by touching the needle at its full retraction to the window membrane (i.e., spider threading mode). Si tend to stick to the needle or tear after a few hundred depositions, whereas SiN films are more robust. In the alternate geometry where droplet and needle do not contact the substrate, threads can be pulled across the width of a microfabricated cantilever substrate and deposited by lowering the tip below the far side of the cantilever (**Figure S4, S5**). A more detailed description of cantilever threading mode is described in **Text S1**.

### IV. Uniformity and straightness

We used fluorescence imaging to assay the angular distribution of m13mp18 threads approximately 20 µm long within two different arrays prepared on the same surface. A least-squares fit measuring thread direction was applied to each of 120 threads taken from four images (**Figure 3A**), which were found to fit a normal distribution with standard deviation of 1.16° for the 20 µm long threads (**Figure 3B**).The angle variability was found to be random across individual threads in the array. This knowledge allows us to estimate the probability that a thread of length *L* will overlap its neighbouring thread at a given thread-to-thread pitch *P*. Using the fitted angular distribution, *L* can be bound by a function of *P* such that crossovers between threads fall within an acceptable error tolerance. To do so, we assume that: (i) the width of a thread is small compared to both thread length and thread pitch and (ii) the distribution of thread-to-surface angles is independent of the thread-to-thread pitch. The location of the first plausible collision between two adjacent threads, one with angle 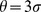 and the other with angle 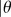, is 
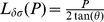
 µm from the edge of the surface, or approximately 8 µm for the 1 µm pitch being examined here. A more typical collision would, for example, involve two adjacent strands at angles 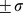, yielding a distance of approximately 25 µm from the surface edge. Inter-thread collisions can typically be avoided for a given pitch by only extracting threads to a conservative 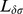 distance, while applications tolerant of overlap may use a more liberal 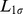 or longer extraction distance.

Besides the angular variation between threads, other types of variations are apparent. These are illustrated in the 12 µm wide area containing ten threads in **Figure 3C** (area sampled from **Figure 3A**), where the intensity profile from the fluorescent image is superimposed onto interval markers representing the programmed thread spacing of ∼1 µm:

1. *Missing threads*: Two of eleven expected threads are missing in the 12 µm window.
2. *Intertwined multiples*: Some threads have higher fluorescence intensity than others, indicating multiple DNA strands.
3. *Forks*: The left-most profile is a pair of threads spaced too closely and which fork toward the bottom of the image, indicating that threads may not always bind the needle at the tip and may interact with each other during extraction.
4. *Lateral deviations from expected thread position*: Threads deviate laterally by up to ∼200 nm in position.

## Discussion

### I. Single vs. multi-strand threads

One consideration for this technique is its ability to deliver single strands versus hairpins or fibrous, multi-strand threads. Based on scanning TEM images from spider threading experiments on thin semiconducting thin films (see **Figure 4A–B** for a representative example), we found strands are often thicker near the needle touch point, and thinner near the droplet meniscus, suggesting that these threads assume a hairpin shape. This observation is consistent with the limited threading success of short DNA in **Figure 2C**. In **Figure 4C**, four threads modified with osbipy label are observed with differing integrated intensities. The background-subtracted relative intensities indicate that the left-most thread comprises about four times as many strands as the two right-most ones, and the second left-most thread comprises about three times as many. Correspondingly, we surmise that, as long as the DNA has a sufficient extraction length, a distribution of single-stranded and multi-stranded segments extending from the meniscus will be present. For applications where single-strandedness is crucial, as in EM-based DNA sequencing, the strands must be very long and extraction lengths scaled commensurately to ensure that the desired quantity of single-strand segments are deposited. At higher EM resolution, quantification of single-strandedness, base-to-base stretching, and observation of individual nucleotide labeling is possible, though this is beyond the scope of this paper and is discussed elsewhere [24], [25]. However, several qualitative features of threading that can be discerned from high-resolution images are examined in **Figure S6**.

### II. Comparison to other linearization techniques

Threaded DNA is straighter than DNA obtained using molecular combing. Threaded osbipy-labeled DNA (**Figure 4D**) deviates laterally by less than 10 nm over longitudinal strand distances exceeding 1 µm, whereas for combed DNA (**Figure 4E**), deviations of the same type are often an order of magnitude larger over the same distances, since variations [20], [31] in the boundary dynamics of the carrier droplet and local interactions between strand and substrate can cause substantial variations in combing quality.

We investigated local roughness and waviness of threads by atomic-resolution TEM, and compared our findings with combing based on several results from the literature [6], [19], [20], [32], [33], [34], [35], [36], [37], [38], [39], [40] (see **Figure 5**). Threads are extremely straight both locally (<100 nm length) and over larger subunit lengths (>100 nm length). In contrast, combed strands are wavy and less uniform even for short subunit lengths (<100 nm length). Combed strands can be very straight in some segments >100 nm long— **Figure 5Ci** exhibits small lateral deviation amplitude of just a few nm—but very large deviations exceeding 10's of nm invariably manifest along the same combed backbone (see **Figure 5Cii**). A survey of molecular combing examples from the literature show that lateral position deviation for favorable combing (straightest and most uniform) is about 120 nm RMS along >10 µm long strand lengths. AFM images at higher magnification show 30 nm RMS fluctuations for shorter (∼1 µm) strand lengths.

Threaded array density is also orders of magnitude higher than combing density (see **Figure 4E**). Threading is typically able to place threads spaced <100 nm apart with >50% yield (a yield of ∼65% is showcased in **Figure 3A**), whereas combing typically places strands 1 µm to 10 µm apart. Moreover, as long as the position of the droplet is continuously adjusted to compensate for evaporation, the same needle can be used to re-thread in a previously-threaded region and thus permits arbitrarily high densities of linearized DNA. Thread yield is related to strandedness through droplet DNA concentration, so lower strand count per thread and high array density are competing parameters; if strand count is not a concern, thread yields of >90% can be obtained by using a sufficiently high (>25 ng/µL) DNA concentration. In contrast, the starting positions of combed strands are defined in stochastic pinning events during the combing process, so that strands are rarely found in high densities. The combing example in **Figure 4E** is a rare case where two combed strands were found very close together and is used for illustrative purposes; adjacent combed strands are rarely separated by less than 10 µm. In surveying the literature, we found combed samples of favorable quality had a density less than 0.01 strands per µm^2^ of sampled image. Angular variability for threaded versus combed arrays was found to be <1° and ∼7°, respectively (<100 µm image field).

Threaded samples are also much cleaner than combed ones since the droplet medium does not contact the array surface. This difference is apparent when comparing threading and combing from the same solution. In combing, uncombed supercoiled DNA balls deposited on the surface during the combing process are typically visible whereas the strand-to-impurity ratio for threading is much higher. However, threading produces hairpin shapes as well as a distribution of strands comprising each thread, so combed DNA is more likely to be single-stranded and fully elongated.

The figures shown, as well as those surveyed in the literature, are selections of high quality threading and combing, although poorly threaded strands may also exhibit imperfections such as kinks, breaks, fibrousness, and gentle curves over micrometer lengths. However, as a general rule, we have found that threading gives more consistent results, orders-of-magnitude improvement in array density, and superior straightness compared to combing at all length scales down to the nanoscale. For DNA linearization, threading also has broader applicability than current AFM techniques. While AFM may currently possess scaling efficiencies by arraying dip pens, these require a pre-patterned surface blueprint for deposition, which limits the number of compatible surfaces [41]. Moreover, for applications such as DNA sequencing or nano-wire patterning where straightness and stretching consistency are priorities, threading is highly suitable.

### III. Future Work

Environmental effects including air currents, droplet evaporation, building vibration, and liquid currents in the droplet can be examined by isolating the apparatus in a sealed, humidity controlled, and/or vibration-damped chamber: their influence on the angular distribution of threads (Figure 3B) as well as other variables can then be quantified. Additionally, the quantitative composition of threads and the degree of stretching in both single- and multi- strand threads are not completely understood. DNA fibres can be directly imaged by TEM when suspended over free space, and we expect that a similar approach will permit direct imaging of unlabeled multi-strand threads [42].

We have observed that threading has two main failure modes as shown in Figure 3C: (i) deposition of multiple threads after a single extraction, and (ii) missing threads. For the first failure mode, the presence of multiple threads can be explained by the tip binding to multiple molecules in the droplet. Alternatively, multiple DNA molecules may interlink in solution (via hybridization, cross-linking, or physical tangling) such that the stretching of one thread bound to the needle induces the stretching and transport of one or more unbound strands. For the second failure mode, missing threads can result from a lack of binding between the needle and DNA, or from a bound molecule that is shorter than the extraction distance being prematurely pulled out of the meniscus. Strategies to reduce the number of failed threads include: (1) mono-functionalized needles to mediate specific interactions with chemically modified DNA and improve binding probability, (2) needles that impose steric constraints on the number of simultaneously bound strands, and (3) tuning the composition of the liquid droplet such that distinct DNA molecules strongly repel one another. Real-time monitoring using fluorescence rather than bright field imaging would also permit direct observation of DNA-needle binding events, from which the time constant for attachment can be inferred.

### IV. Implications of this work and conclusions

The field of DNA nanotechnology has historically been driven by bottom-up, self-assembly techniques [26] but complementary results can often be achieved through high-throughput, top-down nanomanipulation approaches using mesoscale or micro-scale tools. Molecular threading is one such approach which produces reliable results. Because threading stretches DNA in air rather than in liquid, the extended thread can be placed onto water-soluble, dry, or fragile surfaces. Since the positions of threads and the spacings between adjacent threads are determined by the programmed mechanical motion of the piezoelectric nanopositioner rather than stochastic binding events, the user can precisely control the spatial arrangement of threads on the target surface. These advantages are significant for many DNA positioning applications, such as in DNA sequencing by transmission electron microscopy (TEM), where a highly reproducible density of single strands on ultra-thin electron-transparent supports is required [11], [21], [27], [28], [29]. The speed of molecular threading is limited by the motion of the nanopositioner, which can operate with <100 ms cycles. Additional throughput gains may be achieved by threading with arrays of parallel needles. One such scheme is detailed in **Figure S7**.

Applications beyond sequencing include nanofabrication, such as aperiodic templates for organic or inorganic materials using DNA as an organizing scaffold, or precision-patterned DNA nano-wire arrays. Future work will explore the use of molecular threading as a tool for generating new types of DNA nanostructures such as anisotropic DNA sheets or webs, or asymmetric patterns, which have favourable electrical, optical or catalytic properties once modifed. In certain applications, directed overlap between threads – as in Figure 3B for threads stretched more than 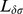 – can be advantageous, and this has been demonstrated in studies involving DNA [30].

Molecular threading shows great potential as a high-throughput DNA manipulation technology. Much work is needed before it can be reliably applied in scaled DNA sequencing or material templating technologies, but in its current form it demonstrates clear advantages over other manipulation techniques such as molecular combing. The straightness, reproducibility, and array density are at least an order of magnitude better than competing techniques. Important next steps include (1) improving the characterization of deposited threads given a range of environmental conditions and (2) improving our understanding of the physics for the DNA unwinding process at the liquid-air interface, which might be achieved through molecular modeling. Answers to these questions would point the way toward a new apparatus design with an eye toward industrial scalability.

## Materials and Methods

### Nomenclature

We term a “thread” to be one or more DNA strands elongated by a PMMA-passivated needle withdrawing from a liquid-air interface and deposited on a surface. A thread must subsequently be interrogated by high resolution imaging to determine whether it is a single strand or a collection of strands.

### Preparation of a DNA-binding Glass Micro-Needle

Micro-needles were pulled from a 1.0 mm diameter borosilicate glass rod using a Sutter P-2000 laser-based micropipette puller with the following parameters: HEAT = 425, FIL = 2, VEL = 20, DEL = 255, PUL = 150. The PMMA suspension was prepared by dissolving 100,000 M.W. PMMA beads (Polysciences) in acetone, and then serially diluting in acetone to a final concentration of 0.1 mg/mL. The tip of the needle was immersed in the suspension for 1 s, then removed from the liquid and suspended in acetone vapor 1 cm above the surface of the PMMA-in-acetone suspension for 1 minute while sealing the container with a gloved hand.

### Instrumentation

Three Sutter MP-225 micro-manipulators were connected to two daisy-chained Sutter MP-200 control boxes and controlled from a single Sutter ROE-200 unit. A PI P-611 NanoCube piezo stage nanopositioner was mechanically coupled to one manipulator using a machined aluminum interface and was connected to a corresponding PI E-664 NanoCube piezo controller. The controller, using a 3× BNC/37 pin sub-D adapter, was connected to an Acces I/O USB-AO16-16A 16-bit analog/digital for control via USB from a desktop computer using software written in Java. Two Qioptiq Optem 70XL micro-inspection lenses were illuminated with two Dolan Jenner MI 150 fibre optic illuminators and were used for real time monitoring.

### Design of component interfaces

A machined aluminum interface for the micro-needle (“needle holder”) connects the micro-needle to the piezo stage. It consists of a fixed aluminum base, attached to the piezo stage, and a removable aluminum needle holder coupled to the base using a spring-clip. A series of grooves, 1 mm in diameter, are cut radially into the needle holder, spanning 0° to −25° at 5° intervals (relative to the plane of the instrument in Figure 1A). A small aluminum block attached to an adjustable screw holds a micro-needle in one of the grooves. In this report, the micro-needle is always oriented at −15°. An image of the interface is shown in Figure S1. An aluminum interface for the substrate (“substrate holder”) was also fabricated. It consists of a base, mechanically coupled to a second micro-manipulator, and a removable platform coupled to the base using a spring-clip. A substrate can be placed directly on the platform. A sample droplet holder formed by a 3/8″ steel rod wrapped in parafilm, was coupled to the third micro-manipulator using a clamp supplied with the device.

### Preparation of substrates

An elastomer gel substrate was removed from a Gel-Pak Gel-Box chip-holder with tweezers, and cut into 0.5 cm^2^ pieces with a razor-blade. A 45° incline was cut into one side of each piece, and each piece was washed in TE buffer for 3 min. A piece was then placed incline-forward on the aluminium substrate holder.

### Preparation of long ssDNA using rolling circle amplification

10 µL of 10× reaction buffer (10× phi29 DNA Polymerase Buffer (B7020, Enzymatics, 500 mMTris-HCl, 100 mM (NH4)2SO4, 40 mM DTT, 100 mM MgCl2, pH 7.5), 1 µL of 1 nM M13mp18 template (NEB), 2.5 µL of 100 nM primer (TCCAACGTCAAAGGGCGAAAAACC, IDT) and 1.6 µL of dNTP mix (Enzymatics N2050L) was brought to a volume of 48 µL in water. The mixture was incubated at 95°C for 1 min, then 60°C for 1 min, then brought to 4°C. The mixture was put on ice, and 2 µL of phi29 DNA polymerase (10 U/µL, Enzymatics P7020-LC-L) was added. The whole mixture was then incubated at 30°C for 4 hr, then brought to 4°C diluted in 450 µL of 1× PBS (pH 7.4). The recovered solution was then diluted 100× in PBS and stored at 4°C until needed. The dilution was brought to room temperature, mixed by pipetting half its volume up and down several times, and then directly used as described in the molecular threading procedure.

### Preparation of long dsDNA molecules

T4GT7 phage stock solution (Wako, 166 kb, 300 ng/µL) was diluted 100× in TE buffer to a final concentration of 3 ng/µL and was stored at 4°C. *λ* phage stock solution (NEB, 48.5 kb, 500 ng/µL), was similarly diluted in TE buffer to a final concentration of 5 ng/µl and was stored at 4°C.

### Molecular threading procedure

A PMMA-treated micro-needle was set at −15° using the needle holder. A cut elastomer substrate was placed with tweezers on the substrate holder. A suspension of DNA molecules was brought to room temperature from 4°C, and mixed by pipetting half its volume up and down several times. A 5 µL droplet was then pipetted on to the end of the parafilm coated rod. To create an array of threads, the piezoelectric nanopositioner executed the following pre-programmed motions:

1. Translate x_1_ µm toward droplet (needle penetrates droplet).
2. Translate x_2_ µm away from droplet (needle and attached thread is withdrawn from droplet).
3. Simultaneously translate x_3_ µm away from droplet and z_1_ µm toward surface (needle contacts surface)
4. Translate z_1_ µm away from surface (needle detaches from thread)
5. Translate y_1_ µm parallel to droplet (needle prepares to thread next element in array).
6. Execute the above instructions N times.

Unless otherwise noted, RCA M13mp18 and T4GT7 phage were prepared with x_1_ = 40, x_2_ = 30, x_3_ = 10, z_1_ = 20, y_1_ = 1 and N = 90, while *λ* phage was prepared with x_1_ = 20, x_2_ = 10, x_3_ = 10, z_1_ = 10, y_1_ = 1, and N = 90.

### Preparation of Surfaces for Fluorescence Microscopy

1 mM YOYO-1 intercalator (Invitrogen) was diluted 10000× in TE buffer (10 mM of Tris-HCl/1 mM of EDTA, pH 8) and a 5 µL droplet was pipetted onto a surface prepared by molecular threading. After a 3 min incubation the surface was washed twice with TE buffer and placed surface-down onto a glass cover slip.

### Fluorescence Microscopy

All fluorescent images were obtained using oil immersion at 160× magnification using a Zeiss Axio Observer Z2 microscope with a Hamamatsu C9100 camera under a GFP filter set (excitation 450–490 nm, emission 500–550 nm). The images presented in Figure 2 were adjusted for brightness and contrast for publication clarity.

### Electron Microscopy

Samples were imaged by electron microscopy in two steps, starting with a screening step at spatial resolutions of approximately 1 nm and followed by high resolution imaging at 1 Å. The screening was conducted in the Hitachi S-5500 - a high-performance scanning electron microscope (SEM) operating in the enhanced-resolution dark field transmission mode (Figure 4). Aberration-corrected scanning transmission electron microscopy (STEM) was carried out in a specialized Nion UltraSTEM 100 [43] modified for automated image acquisition over large sample areas at atomic resolution (Figure 5). Images were automatically captured and post-processed to create composite maps of complete strands. This customized system and its capabilities are described in detail elsewhere [25].

### Image analysis

A line perpendicular to the edge of the substrate surface was superimposed on the left side of each fluorescence image of an array of threads, and a curve was traced on top of each thread in the image. ImageJ [44] was used to obtain a cross-section of each modified image at 1 µm intervals along the measurement axis, and the collection of distances between the measurement axis and the traced curves was used to fit a regression line to each thread. The slope of each regression line was then used to compute the angle made with the measurement axis, and a normal distribution was fit to the distribution of angles. Three outliers were discarded.

Post-processed electron microscopy images were loaded into Gatan DigitalMicrograph, part of the Gatan Microscopy Suite v1.8.0. Straightness and orientation measurements of threaded and combed strands were conducted by placing a reference line for each thread using a least-squares estimate. Maximum deviation measurements were taken along this trendline for rolling 1 µm segments of 100 nm along the reference line. Images of combed samples from the literature used for the comparison in **Figure 5** were first calibrated for scale after loading into DigitalMicrograph and then similarly processed to extract strand angles and lateral deviation figures. Poorly combed strands, such as hooks and loops, were considered outliers and excluded from the averages

## Supporting Information

Figure S1 **The molecular threading apparatus and the primary threading components.** (**A**) The complete apparatus is desktop-sized and relatively portable. (**B**) A small rod covered in parafilm is a sufficient support for the DNA containing droplet which provides the liquid-air interface. (**C**) The angle the pulled-glass micro-needle makes with the droplet surface can be adjusted in 5° increments using a machined holder. (**D**) The PDMS shelf sits on a machined holder; a screw can be turned to align the shelf with the needle and droplet. If a TEM support is placed on the shelf, threads can be directly deposited onto an unsupported thin film.
(TIF)

Figure S2 **Preparation of the working volume using the molecular threading apparatus.** (**A**) Apparatus with primary components detached. The interfaces for the micro-needle, droplet, and substrate holders are each attached to a three-axis long-range micro-positioner. The two cameras used for real time monitoring are positioned over the 120 µm^3^ working volume. (**B**) With the holders attached, the micro-needle, PDMS substrate, and DNA containing droplet are positioned in the working volume. (**C**) Alternate angle of image in (A). (**D**) Alternate angle of image in (B).
(TIF)

Figure S3 **Molecular threading on Si and SiN thin films.** (**A**) Top view. The DNA-containing droplet is brought into contact with the edge of a 5 nm Si unsupported thin film on a TEM grid (TEM Windows). The grid sits on top of the PDMS shelf shown in Figure 1. (**B**) Side view. The micro-needle moves according to Figure **1D** and the description in Materials and Methods. DNA is extracted at the liquid-air interface and deposited when the needle comes into contact with the thin film.
(TIF)

Figure S4 **Design of a cantilever-based substrate for molecular threading.** (**A**): Mock-up of a cantilever substrate. The manipulation handle (**i**) is used as an attachment point for vacuum tweezers for transportation and alignment (with the droplet and micro-needle). The cantilever (**ii**) has several lithographically etched windows across which an unsupported thin carbon film is floated. Any threads deposited across the film can be subsequently examined via transmission electron microscopy. (**B**): An electron micrograph of the cantilever. (**C**): Alignment of the droplet, cantilever, and micro-needle in preparation for threading. Image taken under magnification.
(TIF)

Figure S5 **DRIE/vapor HF processing of cantilever grids.** (**A**) Optical micrograph showing two sizes of cantilever grids fabricated using DRIE/vapor-HF process. Smaller and larger grids are approximately 1 mm×1 mm and 2 mm×2 mm, respectively. Most of the grid is provided for handling and mounting. Sample is threaded across cantilever, perpendicular to long axis. (**B**) SEM image of cantilever from larger grid fabricated by DRIE/vapor-HF. Sample molecules are threaded across cantilever and over circular windows. Windows are approximately 3–4 nm thick and are fabricated with wrinkle-free, amorphous carbon. Inspection at higher magnification showed all windows to be intact.
(TIF)

Figure S6 **High resolution imaging of threads.** (**A**) A representative sample of threaded and labelled DNA used for high resolution imaging. (**B**) Regions of higher intensity along the strand are due to higher label density, likely caused by label aggregation, strand coiling, or cross-linking with small labelled oligonucleotides. (**C**) The needle-tip deposits a region of high label density when transferring its DNA handle to the surface. (**D**) Un-threaded DNA coils, similar to those found in molecular combing, are also deposited during either the alignment or threading process.
(TIF)

Figure S7 **Future work with needle arrays to improve throughput.** (**A**) One scheme to improve throughput via a needle array. (**i**) A “pool” containing long DNA molecules is aligned with an array of glass micro-needles treated with PMMA. A small lateral offset is applied to each row to avoid strand overlap. (**ii**) The entire array is inserted into the solution. (**iii**) Multiple DNA molecules are suspended at the liquid-air interface. A motorized stage supporting a PDMS substrate, or TEM support similar to the cantilever discussed in Text S1, is moved across the surface of the pool in order to collect the extracted strands. (**iv**) The collected strands can now be imaged via fluorescence microscopy or transmission electron microscopy, depending on the substrate. (**B**) Prototype ultra-high-aspect ratio microfabricated needle arrays to be studied in future work. Needle tips have ∼10–20 nm radius of curvature.
(TIF)

File S1 **Microfabrication of “cantilever” EM grids designed for use with threading.**
(DOCX)

Video S1 **DNA Thread Normal to Droplet Surface.**
(MOV)

## References

[1] Bustamante C, Bryant Z, Smith S (2003) Ten years of tension: single-molecule dna mechanics. Nature 421: 423–427. 12540915 10.1038/nature01405

[2] Wilkins M, Gosling R, Seeds W (1951) Nucleic acid: an extensible molecule. Nature 167: 759–760. 14833383 10.1038/167759a0

[3] Bensimon A, Simon A, Chiffaudel A, Croquette V, Heslot F, et al (1994) Alignment and sensitive detection of dna by a moving interface. Science 265: 2096–2098. 7522347 10.1126/science.7522347

[4] Michalet X, Ekong R, Fougerousse F, Rousseaux S, Schurra C, et al (1997) Dynamic molecular combing: stretching the whole human genome for high-resolution studies. Science 277: 1518–1523. 9278517 10.1126/science.277.5331.1518

[5] Herrick J, Bensimon A (1999) Invited review. imaging of single dna molecules: Applications to high-resolution genomic studies. Chromosome Research 7: 409–423. 10560964 10.1023/a:1009276210892

[6] Oshige M, Yamaguchi K, Matsuura S, Kurita H, Mizuno A, et al (2010) A new dna combing method for biochemical analysis. Analytical bio-chemistry 400: 145–147. 10.1016/j.ab.2010.01.021 20085744

[7] Nakao H, Gad M, Sugiyama S, Otobe K, Ohtani T (2003) Transfer-printing of highly aligned dna nanowires. Journal of the AmericanChemical Society 125: 7162–7163. 10.1021/ja034185w 12797774

[8] Smith S, Finzi L, Bustamante C (1992) Direct mechanical measurements of the elasticity of single dna molecules by using magnetic beads. Science 258: 1122–1126. 1439819 10.1126/science.1439819

[9] Lebofsky R, Bensimon A (2003) Single dna molecule analysis: applications of molecular combing. Briefings in functional genomics & proteomics 1: 385–396. 15239885 10.1093/bfgp/1.4.385

[10] Lam E, Hastie A, Lin C, Ehrlich D, Das S, et al (2012) Genome mapping on nanochannel arrays for structural variation analysis and sequence assembly. Nature Biotechnology 10.1038/nbt.2303 PMC3817024 22797562

[11] Cerf A, Alava T, Barton R, Craighead H (2011) Transfer-printing of single dna molecule arrays on graphene for high-resolution electron imaging and analysis. Nano letters 10.1021/nl202219w PMC3205448 21919532

[12] Wang M, Yin H, Landick R, Gelles J, Block S (1997) Stretching dna with optical tweezers. Biophysical Journal 72: 1335–1346. 9138579 10.1016/S0006-3495(97)78780-0 PMC1184516

[13] Tanase M, Biais N, Sheetz M (2007) Magnetic tweezers in cell biology. Methods in cell biology 83: 473–493. 17613321 10.1016/S0091-679X(07)83020-2

[14] Zlatanova J, Lindsay S, Leuba S (2000) Single molecule force spectroscopy in biology using the atomic force microscope. Progress in biophysics and molecular biology 74: 37–61. 11106806 10.1016/s0079-6107(00)00014-6

[15] Cluzel P, Lebrun A, Heller C, Lavery R, Viovy J, et al (1996) Dna: an extensible molecule. Science 271: 792–794. 8628993 10.1126/science.271.5250.792

[16] Greenleaf W, Woodside M, Block S (2007) High-resolution, single-molecule measurements of biomolecular motion. Annual review of biophysics and biomolecular structure 36: 171. 10.1146/annurev.biophys.36.101106.101451 PMC1945240 17328679

[17] Strick T, Allemand J, Croquette V, Bensimon D (2000) Twisting and stretching single dna molecules. Progress in biophysics and molecular biology 74: 115–140. 11106809 10.1016/s0079-6107(00)00018-3

[18] Bensimon D, Simon A, Croquette V, Bensimon A (1995) Stretching dna with a receding meniscus: experiments and models. Physical review letters 74: 4754–4757. 10058590 10.1103/PhysRevLett.74.4754

[19] Allemand J, Bensimon D, Jullien L, Bensimon A, Croquette V (1997) ph-dependent specific binding and combing of dna. Biophysical journal 73: 2064–2070. 9336201 10.1016/S0006-3495(97)78236-5 PMC1181106

[20] Zhang J, Ma Y, Stachura S, He H (2005) Assembly of highly aligned dna strands onto si chips. Langmuir 21: 4180–4184. 15835992 10.1021/la050129s

[21] Andregg W, Andregg M (2010) Sequencing nucleic acid polymers with electron microscopy. US Patent App 12/753,722.

[22] Kanavarioti A, Greenman K, Hamalainen M, Jain A, Johns A, et al.. (2012) Capillary electrophoretic separation-based approach to determine the labeling kinetics of oligodeoxynucleotides. ELEC-TROPHORESIS. 10.1002/elps.201200214 PMC3939315 23147698

[23] Lizardi P, Huang X, Zhu Z, Bray-Ward P, Thomas D, et al (1998) Mutation detection and single-molecule counting using isothermal rolling-circle amplification. Nature genetics 19: 225–232. 9662393 10.1038/898

[24] Own CS, Bleloch AL, Lehrach W, Bowell C, Hamalainen M, et al (2013) First Nucleotide Sequence Data from an Electron Microscopy Based DNA Sequencer. Microscopy and Microanalysis (in press).

[25] Own CS, Lerach W, Elliott L, Bowell C, Stark J, et al In preparation.

[26] Pinheiro A, Han D, Shih W, Yan H (2011) Challenges and opportunities for structural dna nanotechnology. Nature Nanotechnology 6: 763–772. 10.1038/nnano.2011.187 PMC3334823 22056726

[27] Feynman R (1960) There's plenty of room at the bottom. Engineering and Science 23: 22–36.

[28] Bleloch A, Own C, Hamalainen M, Hershleb J, Kemmish K, et al (2011) Dna sequencing by electron microscopy. Microscopy and Microanalysis 17: 1274–1275.

[29] Bell D, Thomas W, Murtagh K, Dionne C, Graham A, et al (2012) Dna base identification by electron microscopy. Microscopy and Microanalysis 18: 1049. 23046798 10.1017/S1431927612012615

[30] Deng Z, Mao C (2003) Dna-templated fabrication of 1d parallel and 2d crossed metallic nanowire arrays. Nano Letters 3: 1545–1548.

[31] Sasou M, Sugiyama S, Yoshino T, Ohtani T (2003) Molecular flat mica surface silanized with methyltrimethoxysilane for fixing and straightening dna. Langmuir 19: 9845–9849.

[32] Cerf A, Thibault C, Geneviève M, Vieu C (2009) Ordered arrays of single dna molecules by a combination of capillary assembly, molecular combing and soft-lithography. Microelectronic Engineering 86: 1419–1423.

[33] Gu Q, Cheng C, Suryanarayanan S, Dai K, Haynie D (2006) Dna templated fabrication of nickel nanocluster chains. Physica E: Low-dimensional Systems and Nanostructures 33: 92–98.

[34] Kim H, Yun D, Choi W, Hong B (2009) Selective assembly of dna using dlc film as passivation layer for the application to nano-device. Diamond and Related Materials 18: 1015–1018.

[35] Kim H, Bae I, Cho S, Boo J, Lee B, et al (2012) Synthesis and characteristics of nh2-functionalized polymer films to align and immobilize dna molecules. Nanoscale research letters 7: 30. 22221314 10.1186/1556-276X-7-30 PMC3275532

[36] Kudo H, Suga K, Fujihira M (2008) Fabrication of substrates with various wettabilities for dna molecular combing. Colloids and Surfaces A: Physicochemical and Engineering Aspects 313: 651–654.

[37] Kwak K, Yoda S, Fujihira M (2003) Observation of stretched single dna molecules by kelvin probe force microscopy. Applied Surface Science 210: 73–78.

[38] Lund J, Dong J, Deng Z, Mao C, Parviz B (2006) Electrical conduction in 7 nm wires constructed on λ-dna. Nanotechnology 17: 2752.

[39] Sagnes M, Broto J, Raquet B, Ondarçuhu T, Laurent C, et al (2003) Alignment and nano-connections of isolated carbon nanotubes. Micro-electronic engineering 67: 683–689.

[40] Schwob E, de Renty C, Coulon V, Gostan T, Boyer C, et al (2009) Use of dna combing for studying dna replication in vivo in yeast and mammalian cells. Methods MolBiol 521: 673–687. 10.1007/978-1-60327-815-7_36 19563133

[41] Nyamjav D, Ivanisevic A (2003) Alignment of Long DNA Molecules on Templates Generated via Dip-Pen Nanolithography. Advanced Materials 15: 1805–1809.

[42] Gentile F, Moretti M, Limongi T, Falqui A, Bertoni G, et al (2012) Direct imaging of dna fibers: the visage of double helix. Nano Letters 12 (12) 6453–6458 (2012). 23171353 10.1021/nl3039162

[43] Krivanek O, Corbin G, Dellby N, Elston B, Keyse R, et al (2008) An electron microscope for the aberration-corrected era. Ultramicroscopy 108: 179–195. 18054168 10.1016/j.ultramic.2007.07.010

[44] Schneider CA, Rasband WS, Eliceiri KW (2012) NIH Image to ImageJ: 25 years of image analysis. Nature Methods 9: 671–675. 22930834 10.1038/nmeth.2089 PMC5554542

