# Supplementary figures and images for: Molecular Threading: Mechanical Extraction, Stretching and Placement of DNA Molecules from a Liquid-Air Interface
Source: PLoS One. 2013 Jul 31;8(7):e69058. doi: 10.1371/journal.pone.0069058 (PMC3729692; doi:10.1371/journal.pone.0069058)

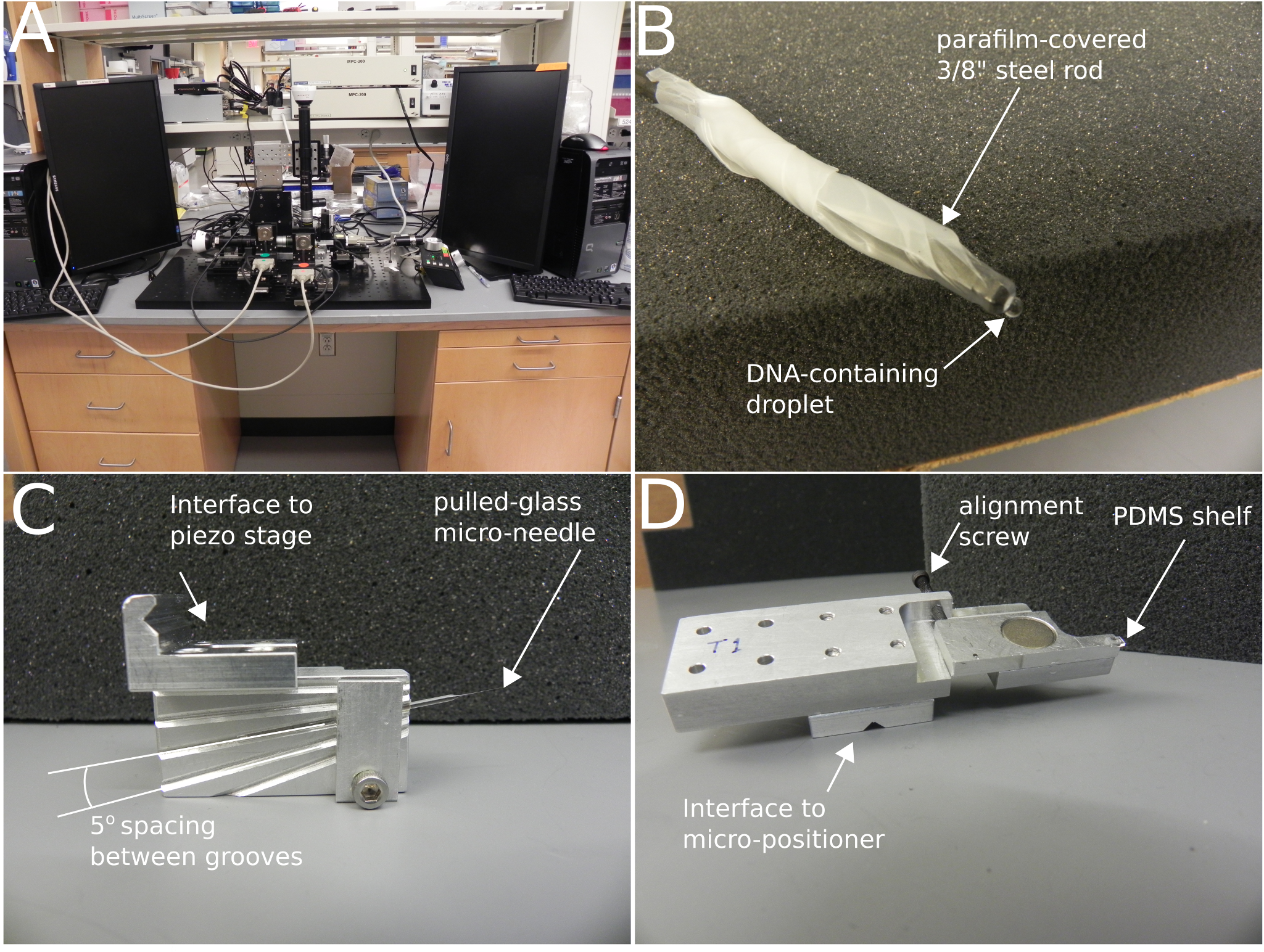

Supplement: Figure S1 — The molecular threading apparatus and the primary threading components. (A) The complete apparatus is desktop-sized and relatively portable. (B) A small rod covered in parafilm is a sufficient support for the DNA containing droplet which provides the liquid-air interface. (C) The angle the pulled-glass micro-needle makes with the droplet surface can be adjusted in 5° increments using a machined holder. (D) The PDMS shelf sits on a machined holder; a screw can be turned to align the shelf with the needle and droplet. If a TEM support is placed on the shelf, threads can be directly deposited onto an unsupported thin film. (TIF) [file pone.0069058.s001.tif]

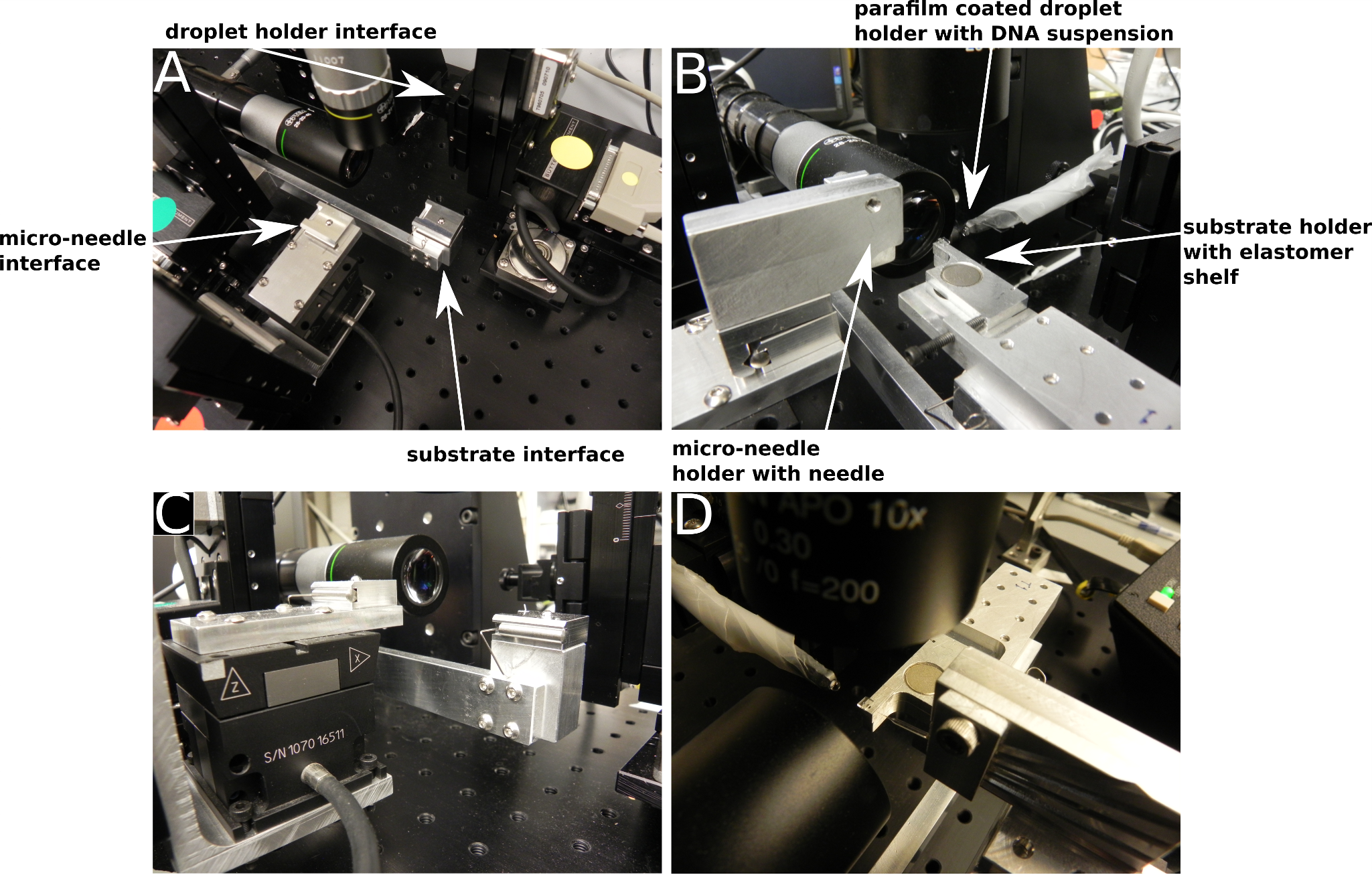

Supplement: Figure S2 — Preparation of the working volume using the molecular threading apparatus. (A) Apparatus with primary components detached. The interfaces for the micro-needle, droplet, and substrate holders are each attached to a three-axis long-range micro-positioner. The two cameras used for real time monitoring are positioned over the 120 µm3 working volume. (B) With the holders attached, the micro-needle, PDMS substrate, and DNA containing droplet are positioned in the working volume. (C) Alternate angle of image in (A). (D) Alternate angle of image in (B). (TIF) [file pone.0069058.s002.tif]

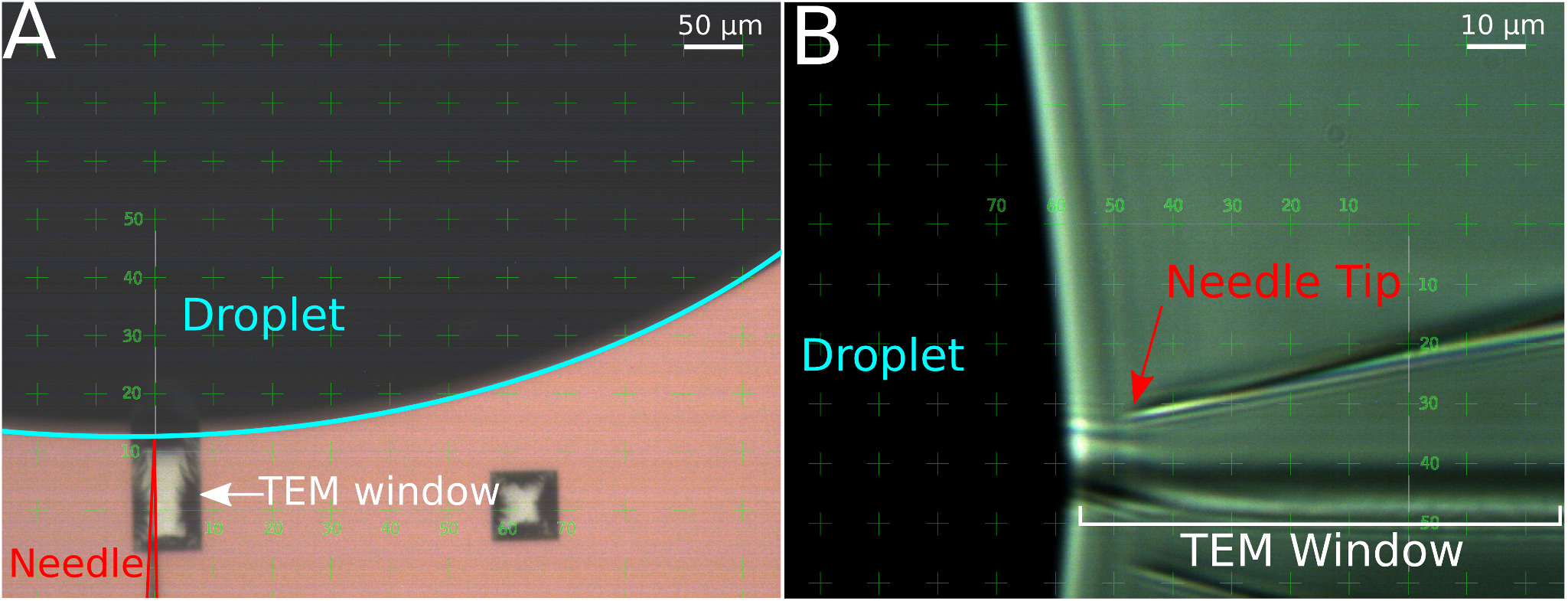

Supplement: Figure S3 — Molecular threading on Si and SiN thin films. (A) Top view. The DNA-containing droplet is brought into contact with the edge of a 5 nm Si unsupported thin film on a TEM grid (TEM Windows). The grid sits on top of the PDMS shelf shown in Figure 1. (B) Side view. The micro-needle moves according to Figure 1D and the description in Materials and Methods. DNA is extracted at the liquid-air interface and deposited when the needle comes into contact with the thin film. (TIF) [file pone.0069058.s003.tif]

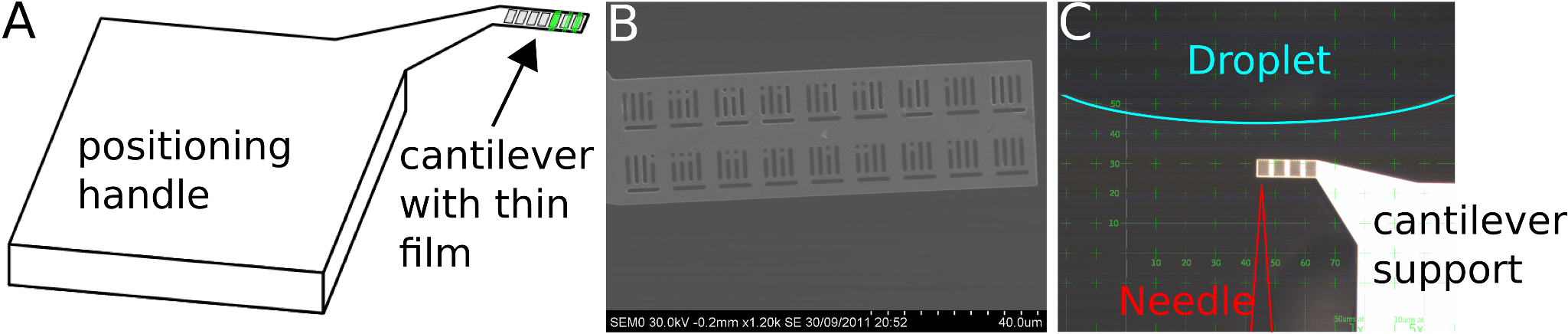

Supplement: Figure S4 — Design of a cantilever-based substrate for molecular threading. (A): Mock-up of a cantilever substrate. The manipulation handle (i) is used as an attachment point for vacuum tweezers for transportation and alignment (with the droplet and micro-needle). The cantilever (ii) has several lithographically etched windows across which an unsupported thin carbon film is floated. Any threads deposited across the film can be subsequently examined via transmission electron microscopy. (B): An electron micrograph of the cantilever. (C): Alignment of the droplet, cantilever, and micro-needle in preparation for threading. Image taken under magnification. (TIF) [file pone.0069058.s004.tif]

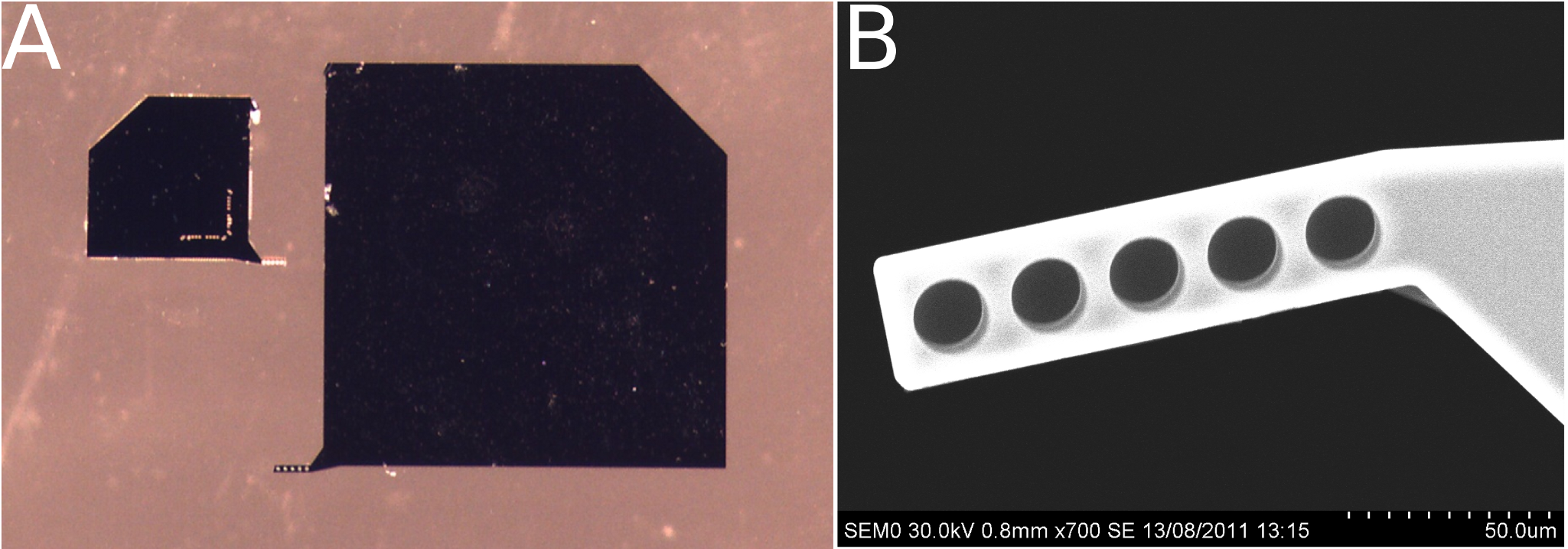

Supplement: Figure S5 — DRIE/vapor HF processing of cantilever grids. (A) Optical micrograph showing two sizes of cantilever grids fabricated using DRIE/vapor-HF process. Smaller and larger grids are approximately 1 mm×1 mm and 2 mm×2 mm, respectively. Most of the grid is provided for handling and mounting. Sample is threaded across cantilever, perpendicular to long axis. (B) SEM image of cantilever from larger grid fabricated by DRIE/vapor-HF. Sample molecules are threaded across cantilever and over circular windows. Windows are approximately 3–4 nm thick and are fabricated with wrinkle-free, amorphous carbon. Inspection at higher magnification showed all windows to be intact. (TIF) [file pone.0069058.s005.tif]

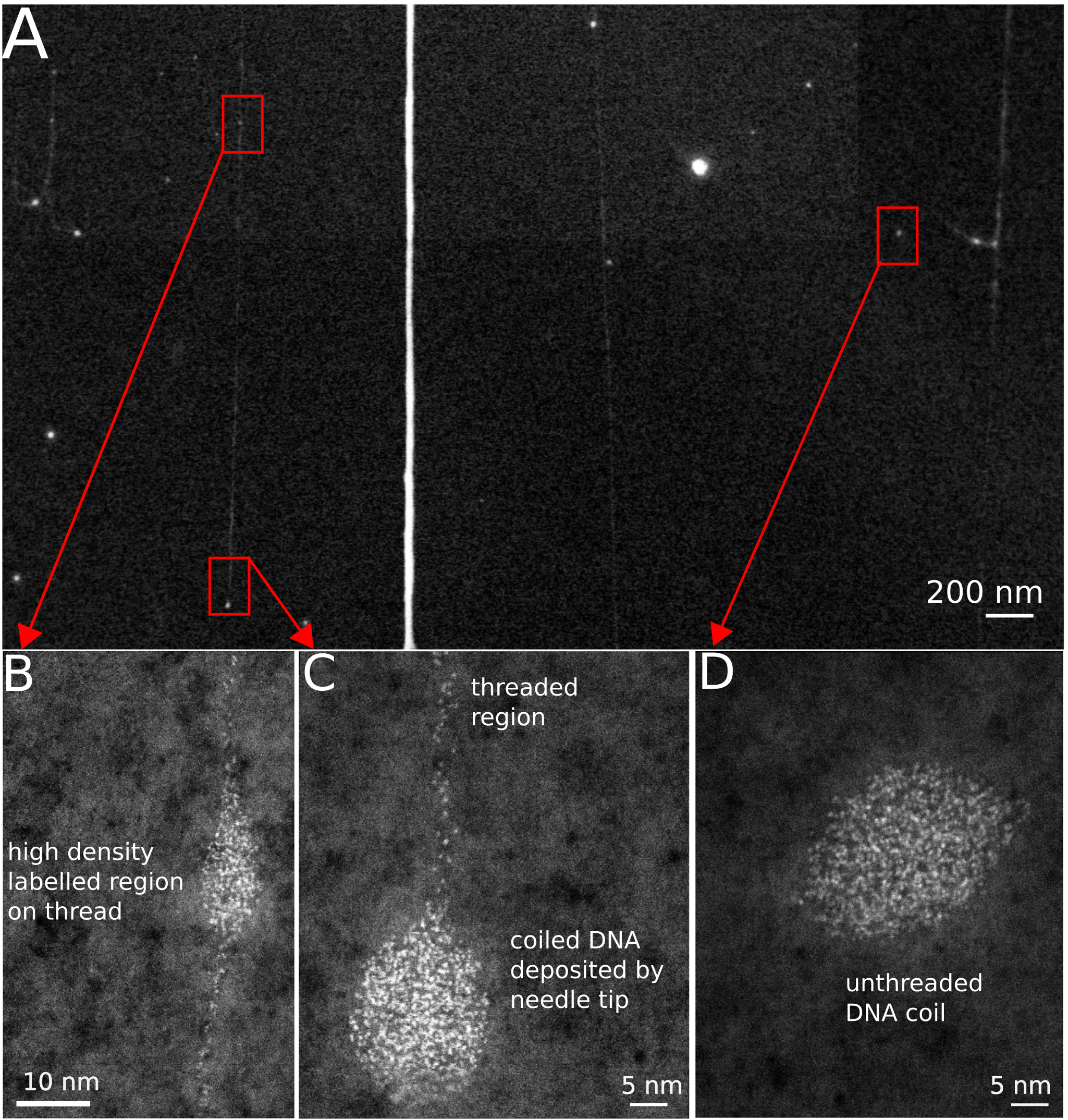

Supplement: Figure S6 — High resolution imaging of threads. (A) A representative sample of threaded and labelled DNA used for high resolution imaging. (B) Regions of higher intensity along the strand are due to higher label density, likely caused by label aggregation, strand coiling, or cross-linking with small labelled oligonucleotides. (C) The needle-tip deposits a region of high label density when transferring its DNA handle to the surface. (D) Un-threaded DNA coils, similar to those found in molecular combing, are also deposited during either the alignment or threading process. (TIF) [file pone.0069058.s006.tif]

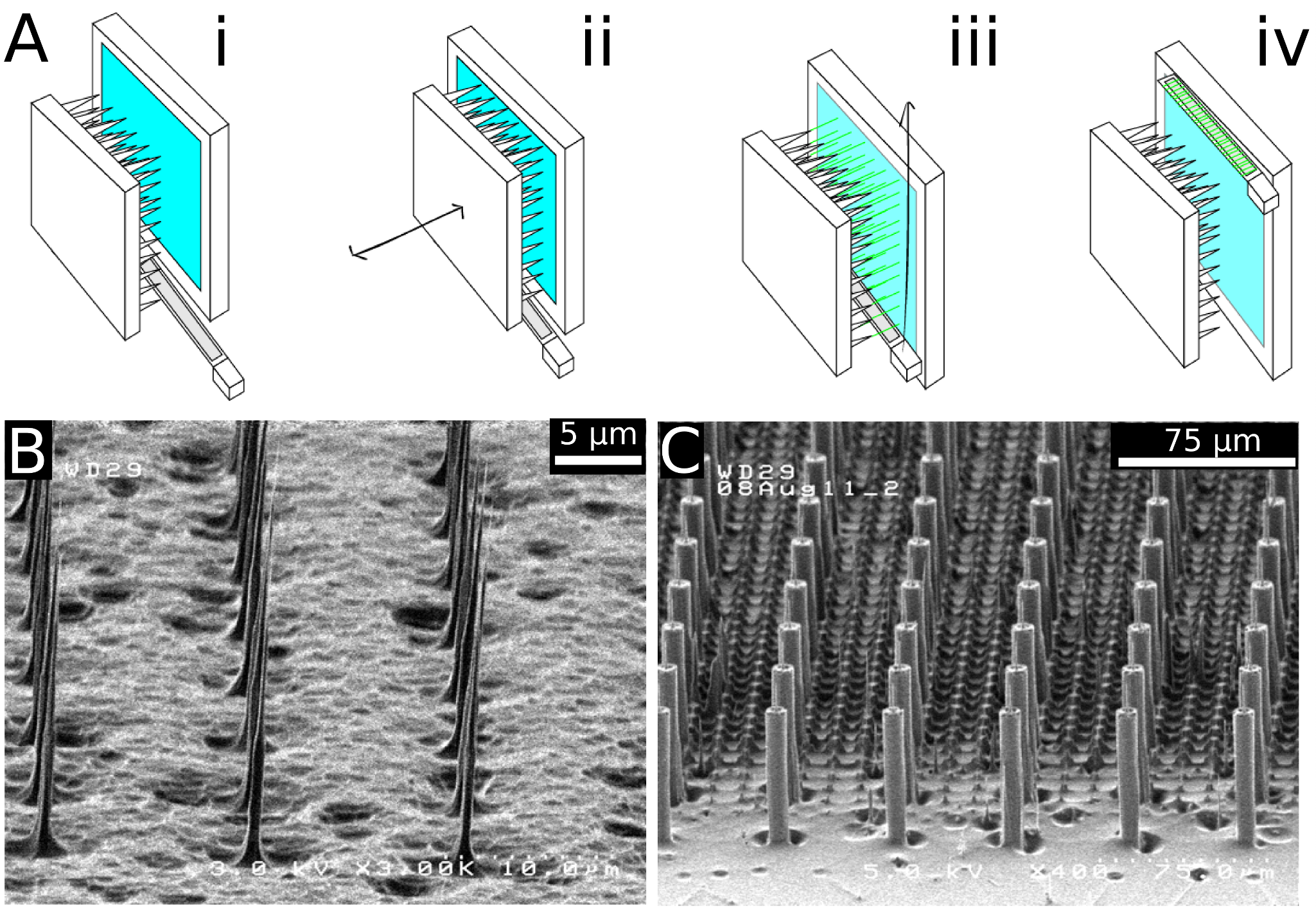

Supplement: Figure S7 — Future work with needle arrays to improve throughput. (A) One scheme to improve throughput via a needle array. (i) A “pool” containing long DNA molecules is aligned with an array of glass micro-needles treated with PMMA. A small lateral offset is applied to each row to avoid strand overlap. (ii) The entire array is inserted into the solution. (iii) Multiple DNA molecules are suspended at the liquid-air interface. A motorized stage supporting a PDMS substrate, or TEM support similar to the cantilever discussed in Text S1, is moved across the surface of the pool in order to collect the extracted strands. (iv) The collected strands can now be imaged via fluorescence microscopy or transmission electron microscopy, depending on the substrate. (B) Prototype ultra-high-aspect ratio microfabricated needle arrays to be studied in future work. Needle tips have ∼10–20 nm radius of curvature. (TIF) [file pone.0069058.s007.tif]
