# Supplementary material for: Molecular Threading: Mechanical Extraction, Stretching and Placement of DNA Molecules from a Liquid-Air Interface
Source: PLoS One. 2013 Jul 31;8(7):e69058. doi: 10.1371/journal.pone.0069058 (PMC3729692; doi:10.1371/journal.pone.0069058)
Supplement: File S1 — Microfabrication of “cantilever” EM grids designed for use with threading. (DOCX) [file pone.0069058.s008.docx]

# Molecular threading: mechanical extraction, stretching and placement of DNA molecules from a liquid-air interface

## Supplementary Information

Andrew C. Payne^1,2^*, Michael Andregg^2^*, Kent Kemmish^2^, Mark Hamalainen^2^, Charlotte Bowell^2^, Andrew Bleloch^2^, Nathan Klejwa^2^, Wolfgang Lehrach^2^, Ken Schatz^2^, Heather Stark^2^, Adam Marblestone­^3^, George Church^3,4^**^†^, Christopher S. Own^2^**^‡^, and William Andregg^2^

**1** Wyss Institute, Harvard University, Boston, Massachusetts, United States of America, **2** Halcyon Molecular, Inc., Redwood City, CA, United States of America**, 3** Biophysics Program and Wyss Institute, Harvard University, Boston, Massachusetts, United States of America, **4** Department of Genetics, Harvard Medical School, Boston, Massachusetts, United States of America

**Text S1. Microfabrication of “cantilever” EM grids designed for use with threading**

Threading of DNA provides a means for placing multiple, parallel aligned, and closely spaced strands on a substrate. In addition, the deposited strands have been found to have more consistent base spacing and straightness on the nano- and micro-scale than combed DNA. These attributes make threading an excellent technique for preparing DNA samples for electron microscopy, and in particular for experiments with DNA sequencing by EM.

Two types of EM grids were designed for use with threading and made using MEMS microfabrication techniques. A first type provides a cantilever-shaped frame onto which a 3-5nm thick carbon film is floated **(Figure 4)** [45]. An improved design that avoids the issues associated with a floated film uses a cantilever-shaped frame, defined by DRIE etching, with 50-100nm thick silicon dioxide windows. A 3-5 nm thick amorphous carbon film is deposited over the top of the cantilever and windows, and using a HF-vapor etching process [46], the silicon dioxide windows are etched away leaving wrinkle-free thin carbon windows. **Figures S5 (A)** **and (B)** show optical and SEM images of grids fabricated using a combination of DRIE and vapor-HF etching.

Samples are prepared by threading DNA across the width of the cantilever. To avoid damage to the thin carbon film during threading, the sample-containing droplet and needle are not allowed to touch the cantilever, and hence the arrangement is slightly different from that depicted in Figure 1. While threading over a microfabricated grid, the sample-containing droplet is positioned approximately 10 $\mu$m from the near side of the cantilever, and the needle trajectory is adjusted such that at full retraction, the tip of the needle is past the far side of the cantilever and below the top surface of the cantilever. With such a needle trajectory, the DNA is lowered down onto the cantilever surface without the needle contacting the cantilever. Sample strands must be long enough to stretch from the droplet to the fully retraced needle-tip, and hence the minimum strand length is approximately 50 $\mu$m for 30 $\mu$m wide cantilevers. T4 GT7 DNA (Wako Chemicals USA, Inc., produced by Nippon Gene), at 167kbp long, has been successfully threaded onto 30micron wide cantilevers.

An outline of the process used to fabricate grids with HF-vapor etched sacrificial silicon dioxide windows is as follows:

1. Material: Silicon wafer, <100>, 100mm dia, >1 ohm-cm, 50 $\mu$m thick, double-sided polish.
2. Wet Clean, standard oxidation-furnace pre-clean – wet cleans require special wafer holder to prevent wafers from floating to top of bath (due to bubbling of solutions, wafer movement and floating in piranha etch is troublesome).
3. Grow 100nm thermal oxide (TOX).
4. Pattern backside-alignment-marks into backside of wafer using lithography and plasma etching, etching through TOX and into Si a few $\mu$m.
5. Pattern TOX on front-side of wafer, aligning to backside-alignment-marks. Requires mask-aligner or other lithography tool with backside or IR alignment capability. Patterning leaves TOX disks which will become sacrificial TOX windows after DRIE
6. Bond wafer in vacuum oven, device-side down against full thickness handle wafer with Waferbond CR-200 (Brewer Science). Bonded handle wafer improves heat transfer during DRIE etch, thereby minimizing footing under TOX windows and preventing wafer breakage.
7. Pattern inverted grid layout on backside of wafer, aligning to backside-alignment-marks.
8. Etch through TOX on backside of wafer, then DRIE etch through wafer, stopping on device-side TOX windows
9. Remove grids from handle wafer by soaking in heated Waferbond Remover (Brewer Science), followed by 3x IPA rinse.
10. Clean grids by soaking in ethanolamine, 110°C for 20min, 3x DI rinse, followed by SC-1 for 40min, 5x DI rinse, and vacuum bake 600°C, 10hrs.
11. Deposit 3-5nm amorphous-carbon onto device side of grids using an arc evaporator (Cressington 208C with turbopump).
12. Remove TOX sacrificial windows using HF-vapor, 90 minutes at 50C in heated aluminum holder located immediately above, but not sealed to, mouth of 500ml bottle of 49% HF. HF bottle remains at room temperature, only holder and grids are heated. (HF warning. Receive HF training and ware PPE designed for HF use. Process must be conducted in fume hood.)
13. Final clean – within a few hours of threading, place grid directly on UV filter of 20W halogen lamp for 20 min.

An outline of the process used to fabricate cantilever grids for use with floated carbon follows. The main issue to note with this process is that wafer integrity must be maintained throughout the DRIE etch. This is achieved by balancing stress on the top and bottom of the wafer, which requires that the thickness of oxide on the backside of the wafer approximately equal the sum of the BOX and top surface oxide thickness.

1. Material: Silicon SOI bonded wafer, <100>, 100mm dia, 7 $\mu$m device, 1 $\mu$m BOX, 250 $\mu$m total.
2. Wet Clean, standard oxidation-furnace pre-clean
3. Grow 1.7 $\mu$m TOX.
4. Strip TOX from device-side of wafer, leaving TOX on backside.
5. Grow 1.0 $\mu$m TOX (device-side, total TOX on backside should be approximately 2um).
6. Pattern device-side of wafer and etch through device-side stopping on BOX.
7. Protect device-side by coating device-side of wafer with thick photo resist and hard bake.
8. Pattern backside of wafer with thick photo-resist. Hard bake for maximum erosion resistance.
9. Etch through TOX on backside, and DRIE backside of wafer, stopping on BOX.
10. Strip PR with acetone, then piranha clean.
11. Strip TOX from device and backside of wafer and remove exposed BOX with HF.
12. Deposit 3-5 nm carbon on freshly cleaved salt or mica, dip grid in HF to make surfaces hydrophobic, place grid device-side down on carbon, float off carbon into water (grids will float off with carbon), recover grids from water.
13. Final clean – within a few hours of threading, place grid directly on UV filter of 20W halogen lamp for 20 min.

**References**

[45] Johansen B (1974) Bright field electron microscopy of biological specimens. ii preparation of ultra-thin carbon support films. Micron (1969) 5: 209-221.

[46] Witvrouw A, Du Bois B, De Moor P, Verbist A, Van Hoof C, et al. (2000) A comparison between wet hf etching and vapor hf etching for sacrificial oxide removal. In: proc. SPIE. volume 4174, pp. 130-141.
